# Can the Public–Private Business Model Provide a Sustainable Quality Pediatric Cardiac Surgery Program in Low- and Middle-Income Countries?

**DOI:** 10.1177/21501351221151057

**Published:** 2023-02-14

**Authors:** Stephany Kim, Sreemathi Seshadrinathan, Kathy J Jenkins, John S Murala

**Affiliations:** 1 Department of Cardiovascular and Thoracic Surgery, University of Texas Southwestern Medical Center, Dallas, TX, USA; 2 Department of Cardiovascular and Thoracic Surgery, Public Health Centre, Chennai, Tamil Nadu, India; 3 Department of Cardiology, Boston Children's Hospital, Boston, MA, USA

**Keywords:** congenital heart surgery, congenital heart disease (CHD), health policy (includes government regulation), international collaboration

## Abstract

Over 90% of the world's children with congenital heart disease do not have access to cardiac care. Although many models provide pediatric cardiac surgery in low- and middle-income countries, sustainability poses a barrier. We explore one model providing care for the underserved in Chennai, India, that came into existence through trial and error over 30 years across three phases. Phase 1 was a Tamilnadu state government–sponsored program that soon became unsustainable with unmet demands. Phase 2 utilized a grassroots foundation of a public–private partnership (PPP) with few donors and a hospital with suboptimal infrastructure. Phase 3 is the ongoing fine-tuning of the PPP model, with upgraded infrastructure and a well-trained team. Through indigenization, an average cardiac surgery costs Rupees (Rs.) 1,80,000 ($2400). The government funds Rs. 60,000 to 80,000 ($800-$1066.67), and the rest is funded through the fund pool. The goal is to perform 100 free surgeries annually by maintaining a fund pool of Rs. 50 lakhs ($66,666.67), which supplements government funds. This ensures equitable distribution of funds with no compromise on resources (disposables, single-use cannulas, etc). Our model ensures the dignity of the patient, fair compensation for workers, and is practical, affordable, and easily adaptable. Thus far, this model provided free cardiac surgery for 357 children from Risk Adjusted Congenital Heart Surgery Score of 1 to 4, with an overall mortality of 2.73%. The prerequisites for this model are having a “spark plug,” a dedicated surgical team, a partnership with state-of-the-art infrastructure, and a steady flow of funds.

## Introduction

It is postulated that there are over 100 billion humans who have ever lived on Earth.^1^ The global population is presently nearing 8 billion and will grow exponentially to 9.7 billion by the year 2050 in 244 entities, which include countries, territories, colonies, and dependencies, with major growth in low- and middle-income countries (LMICs), especially the Sahel and sub-Saharan Africa.^2^

Although efforts to better deliver affordable cardiac care to populations in LMICs have improved, glaring disparities continue to exist. There is the imbalance when comparing the burden of the absolute number of cardiac surgical patients, which is a hundred-fold greater in many LMICs in Asia and Africa compared to North America and Europe.^3,4^ Diseases such as rheumatic heart disease are common in LMICs, which are compounded by limited resources, whereas such diseases are a rarity in high-income countries.^5^ The rise in noncommunicable diseases (NCDs), such as cardiovascular disease, reflects a global shift toward increasing life expectancy and nutrition. Additionally, urbanization has worsened already existing disparities in places with uncorrected poverty.^6^ LMICs are not only disproportionally affected by NCDs but they also continue to face the burden of communicable diseases. In juggling the “double disease burden,” their health care systems are overwhelmed.^7^

Congenital heart disease (CHD) affects 1 in 110 children.^8^ An estimated 300,000 people die annually in LMICs due to lack of cardiac care and untreated CHD.^9^ Currently, over 70% of the facilities reach less than 20% of the world's population, leaving over 90% of children born with CHD without any access to cardiac care.^10^ As such, it is evident that the global burden of cardiac care is heavily skewed and LMICs continue to remain underserved.

## Background

Many cardiac programs in LMICs have attempted to provide free or highly subsidized care to vulnerable populations with government funding, which have often fallen short of a sustainable program.^11^ There is also inadequate investment in healthcare as indicated by 2019 World Health Organization (WHO) data, wherein India only spent 3% of total gross domestic product on health as compared to 17% spent by the United States.^12^ In addition, among many households in LMICs, there is often low willingness to pay for health care insurance.^13^ Therefore, with limited government funding and personal financing/insurance, cardiac care in LMICs needs alternative financing models, especially with limited resources, such as conduits, bypass disposables, suture materials, and prosthetics.^14^ One way in which programs manage to drive the costs down is by reusing disposables and outsourcing near-expired resources.^15,16^ The problem inherent to this model is that programs save precious resources, but at the cost of true dignity for the patient. As such, in the proposed model, our goal is to find a way to finance programs using the same material for free for nonpaying patients as is used for normal-paying patients.

Currently, there are several types of financing mechanisms for programs that provide free cardiac care for the underprivileged. Some of them include the private or co-financing model, the public–private partnership (PPP), fiscal space expansion, and development aid for health.^17,18^ The private or co-financing model involves frugal innovation, which is defined by Zeschky et al as “good-enough, affordable products that meet the needs of resource-constrained consumers.”^19^ Narayana Hrudayalaya Hospital in Bengaluru, India, is currently built on the cross-subsidization model in the private/co-financing sector.^20^ They can achieve this because of economies of scale or the conveyer belt model and frugal innovation.^21,22^ The PPP is another model, one which we utilized in our business model. The PPP model has gained some traction in countries like India and has been hailed as a “clever policy to utilize the excellence of the booming private tertiary care centers in India for the benefit of the poor.”^23^ This is mutually beneficial and the addition of government-sponsored patients will help in sliding scale payments.^11^ The other models are fiscal space expansion, which refers to the capacity in a government's budget to provide additional resources for health without jeopardizing its long-term economic stability,^17^ and development aid for health, which includes external funds transferred to LMICs from major development agencies. Asides from the private model, the other three models have one thing in common: they are dependent on the government for funds. Unfortunately, there are many LMICs where government funding does not exist at all.

The above models do not include religious organizations and foundations that provide free health care. Although affiliations with nongovernmental organizations (NGOs) have previously utilized surgical volunteerism as a common platform, our goal is to model by innovation, which is essential for improvements in the health care system, both in terms of product and processes.^24^ When charity organizations and NGOs provide transient care (often short-term missions) to patients in LMICs, the costs are steep, benefits reach only a select number of patients, and by and large there seems to be a suboptimal transfer of skills to sustain a viable cardiac program.^10,25^ This necessitates a deeper involvement and development of partnerships between health care infrastructures, governmental institutions, and communities.^26^ Although “twinning programs” between a seasoned partner from elsewhere and a partner from an LMIC is still a useful strategy, we hope that a local partnership between a public and private institution will provide us with novel opportunities to support and sustain cardiac programs in some LMICs. We do acknowledge that this may not be possible in many LMICs, where there are limited government resources, and where short-term missions may be the only practical means.

## Methods

This is an observational study with some retrospective data of a PPP model developed by one of the authors (SS). It was achieved over three decades of trial-and-error and includes her lifetime efforts across three phases. This model has a “spark plug,” which we identified previously as being one of the essentials for building a successful program in LMICs.^10^ Only de-identified data were used; hence, our institution determined this project to be a nonhuman research project and did not require Institutional Review Board processing. The aim of this study is to describe the various phases of evolution of the present PPP model, whose goal is to provide high acuity, pediatric cardiac surgery free of cost to economically challenged children in Chennai, India. The current PPP model is a result of progressive development from three unequally spaced phases starting from 1988 across three different hospitals. Of note, the currency conversion rate used is 1 United States Dollar ($/USD) ∼ 75 Indian Rupees (Rs) from March of 2022.^27^

### The Evolution Over Three Stages

**“**Considering a birth prevalence as 9/1000, the estimated number of children born with CHD every year in India approximates 240,000, posing a tremendous challenge for the families, society and health care system.”—Dr Anita Saxena^28^

### Phase 1 (1988-2002)

**Figure 1.**
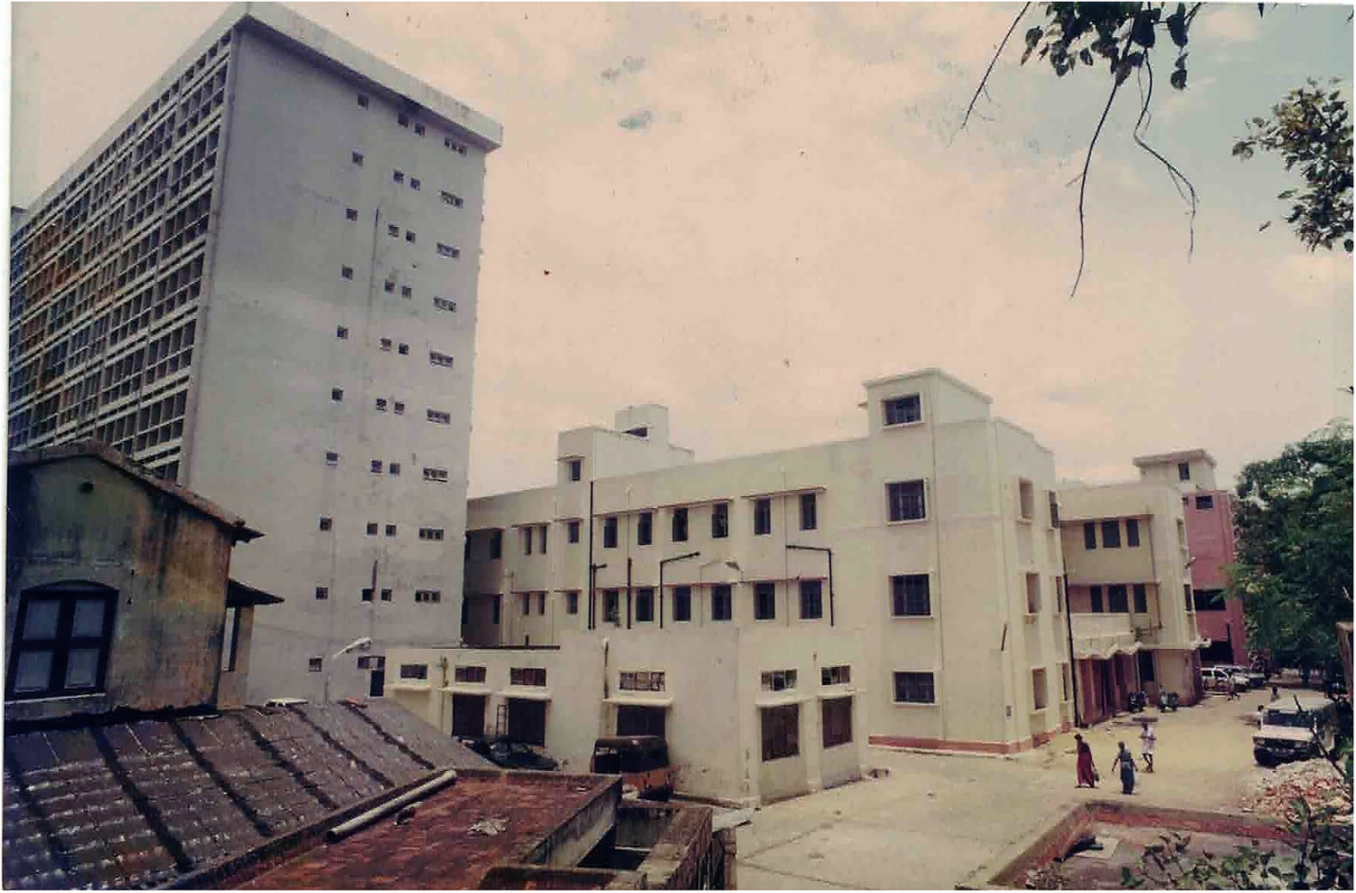
Picture of the Institute of Child Health and Hospital for Children in Chennai, India.

**Figure 2.**
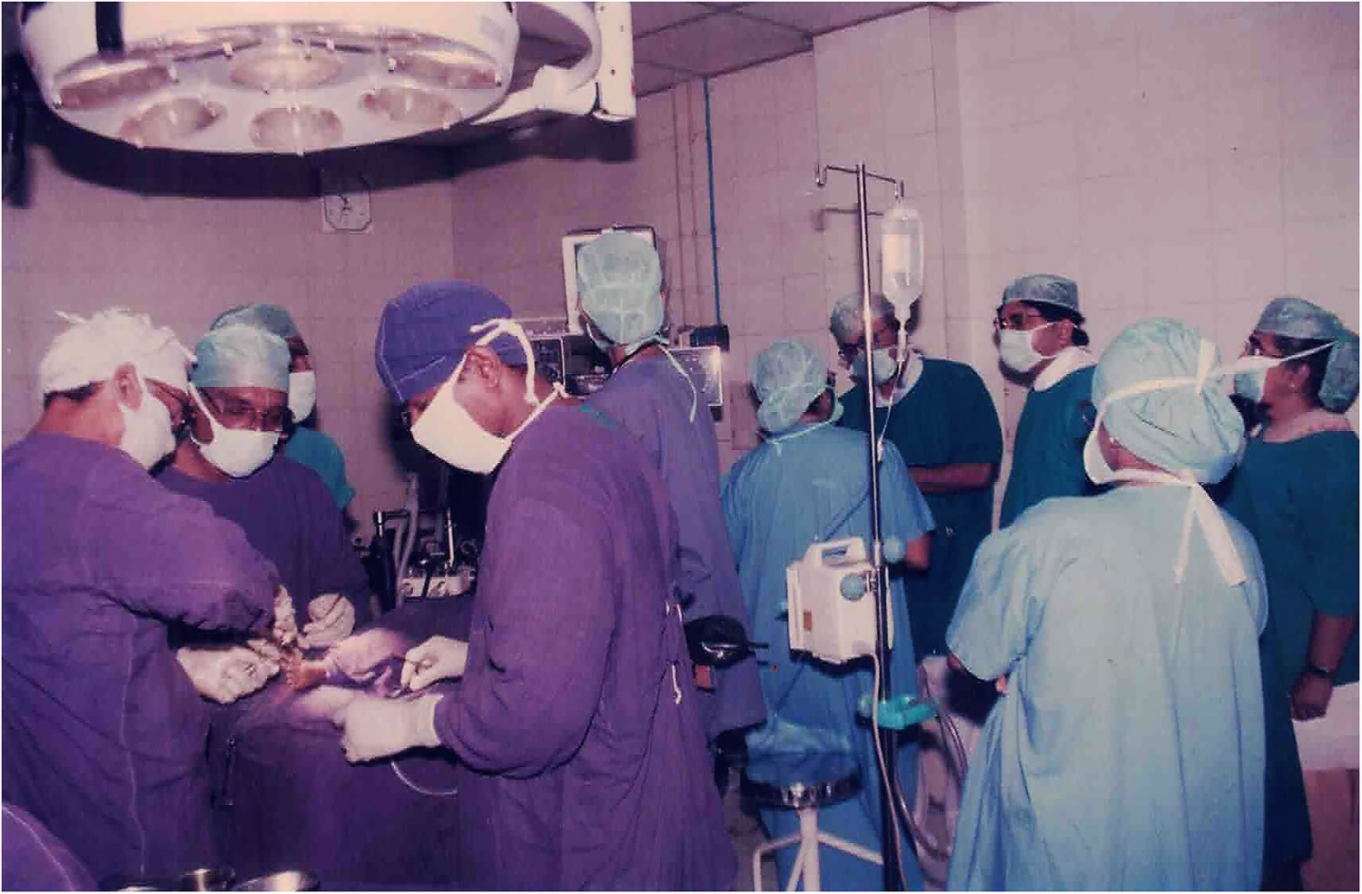
Operating theater with surgical team at the Institute of Child Health and Hospital for Children.

Initially, the Institute of Child Health and Hospital for Children in Chennai, one of the largest hospitals for children in India, provided the infrastructure to perform surgeries (Figure 1). Funding for this project was from the state and central governments, the World Bank, and the government of Japan. The hospital had reasonable infrastructure and the capacity to perform four surgeries per day (Figure 2). Over 5732 surgeries were performed with gross mortality of 12% in the initial stages (1988-1990), which decreased to 3.92% in the latter half (1990-2002).

Many children were malnourished, anemic, and hypoproteinemic, which also contributed to the mortality in the initial stages. Change in strategy to address nutrition and anemia perioperatively improved the outcomes and survival in the second stage when more complicated surgeries were performed. Despite the success, 700 children from lower socioeconomic groups awaited surgery with increased waitlist mortality. The challenges were due to limited resources, rising demand, a surgical team on the learning curve, and limited government funds.

### Phase 2 (2002-2013)

The second stage began with the early foundations of the PPP model. The surgical team remained the same. Dr SS helped form a three-way partnership between the Indian government, a charitable trust, and a private hospital (K.J. Hospital Research & Postgraduate Centre in Chennai). Although this multisource funding supported the project, this would soon become a major hurdle. The infrastructure was suboptimal, which affected the quality of care. Risk Adjusted Congenital Heart Surgery Score (RACHS) was used to determine the complexity of cases.^29^ RACHS 1, 2, 3, and 4 surgeries were routinely performed. However, the mortality rate remained at 3.92% for the total of 187 free surgeries performed during this phase. This stage emphasized the importance of a trained team in exchanging knowledge, improving surgical skills, and focusing on the quality of care while underscoring the drawbacks of working with suboptimal hospital infrastructure. Significant advancements at the end of the second phase included the introduction of the program to the International Quality Improvement Collaborative for Congenital Heart Disease (IQIC) from Boston Children's Hospital and partnership with Dr William Novick's team, which contributed to the exchange of ideas (eg, double-flap method of closure of ventricular septal defect with pulmonary hypertension^30^).

This phase also witnessed the landmark government insurance scheme called the Rajiv Arogya Shri program that was launched in the state of Andhra Pradesh in 2007.^31^ Soon, the state of Tamil Nadu also adopted a similar government insurance scheme to cover heart surgery.

### Phase 3 (2013 to date)

**Figure 3.**
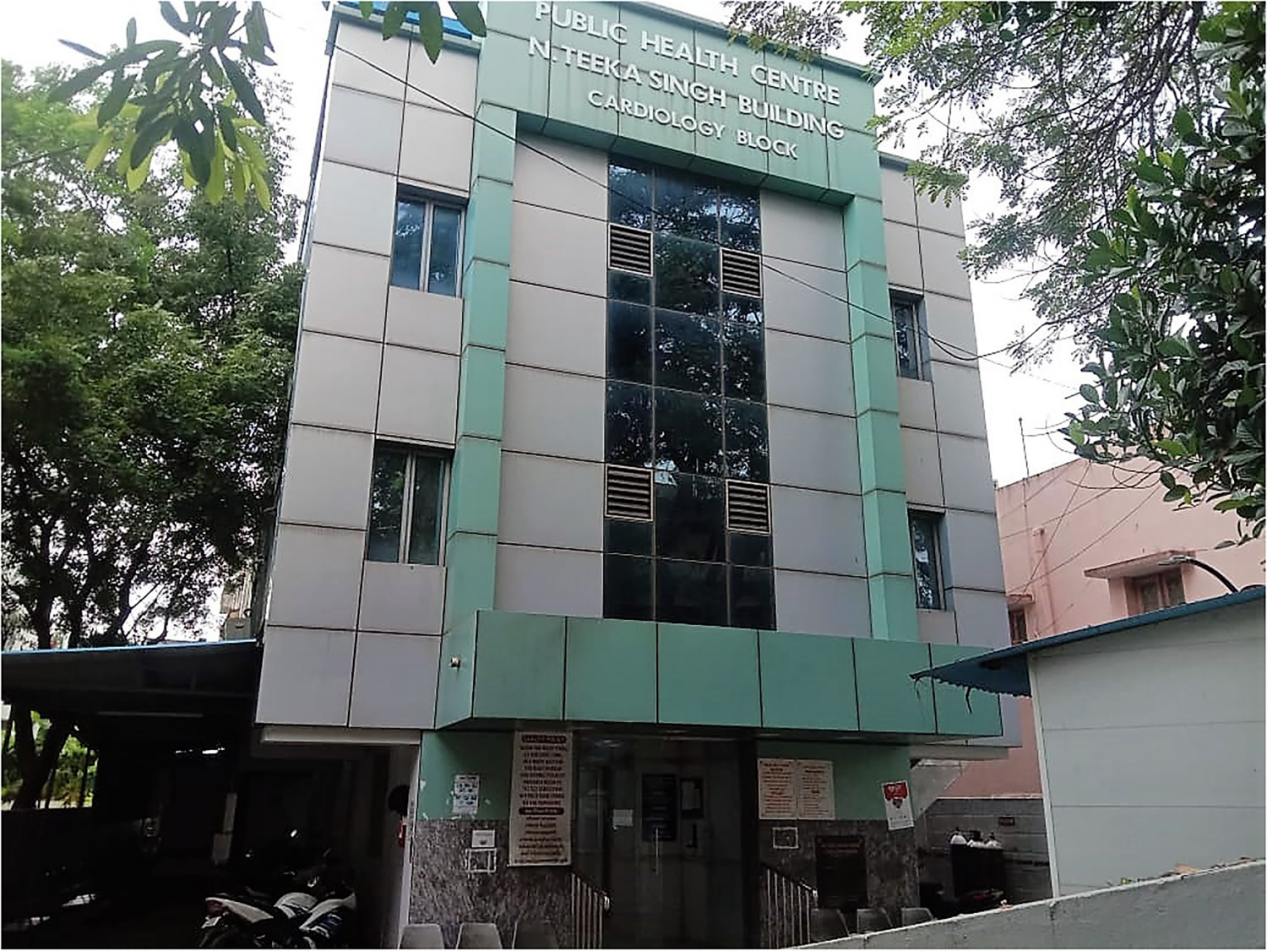
Picture of the Public Health Centre Hospital.

With the lessons learned from the first two phases, the PPP model continued with the partnership with Public Health Centre Hospital (PHC), a wing of an autonomous nongovernmental Public Health Care Society (Figure 3). In collaboration with PHC, the Hearts 4 Hearts (H4H) fund was founded. This expansion of the fund allowed surgeons, anesthesiologists, perfusionists, and nurses to be paid on a case-by-case basis, while PHC provided income to the intensive care unit (ICU) nurses employed by the hospital. The present team consists of qualified, trained, and experienced medical professionals, nurses, and paramedical staff. Additional support is provided by the following:

1. Udhavum Karangal, a home for the destitute, partnered with our program and provided accommodation and food for patients and family during surgery and follow-up visits.
2. The Chief Minister's Comprehensive Health Insurance Scheme (CMCHIS) provided a network of referral doctors in various districts across India.
3. Government funds financed the hospital, while intermittent funds from donors, crowdfunding, and Solution for Cardiac Affected Needy (SOCAN) supplied the rest. SOCAN is a holistic project under H4H.
4. A network of recurrent doctors filled the gaps due to expenses in the fund pool in H4H.
5. Continued collaboration with Boston Children's Hospital and contributions toward the IQIC database helped in quality control and empowerment of the local team.

All the above partnerships help create cost-effective care while maintaining high quality. A total of 170 free surgeries were performed in this phase with an overall (RACHS 1-4) mortality of 2.73% and mortality rate of <1% for RACHS 1 and 2 categories (see Table 1 for mortality across all three phases). This was possible by improving postoperative ICU care and empowering nurses.

**Table 1.** Gross Mortality of Pediatric Cardiac Patients Across All Phases.

Phase	Gross mortality %	Morbidity %
Phase 1	12% to 3.92%	14% to 5%
Phase 2	3.92%	5%
Phase 3	3.92% to 2.73%	5% to 2%

To strengthen the financing aspect of the model, we created a system for donors and organizations by categorizing them into levels of donations. The four levels were less than Rs. 10,000 (<$133), between Rs. 10,000 and Rs. 1 lakh (Rs. 1,00,000) ($133-$1333.33), between Rs. 1 lakh and Rs. 5 lakhs ($1333.33-$6666.67), and greater than Rs. 5 lakhs (>$6666.67). Additionally, a feedback system provided the donors opportunities to receive regular updates on the beneficiary's progress, which further incentivized donors and personalized their donations. From here, the idea of a fund pool was born. This would require a minimum of Rs. 50 lakhs ($66,666.67). The fund pool would sustain a minimum of 30 to 40 free surgeries simultaneously, and any shortfall could be covered by government funds or recurrent donors. The revolving fund will cater to differences in expenses for various surgeries and in time will become self-supporting and sustaining. Numbers in this phase have been small due to the small fund pool. The goal is to actively increase the fund pool size in stages, with the ultimate goal of creating a corpus fund.

### Finances and Cost Analysis

The ideal goal is to perform 100 free surgeries per year at PHC. Here, we would like to clarify that the hospital also routinely operates on paying patients. Through safe measures and indigenization, an average cardiac surgery (after the onset of the COVID pandemic) costs Rs. 1,80,000 ($2400) in this model. Funds raised from CMCHIS and recurrent donors total Rs. 1,10,000 ($1466.67) per surgery, which is still short Rs. 50,000 ($666.67) (pre-COVID) and Rs. 70,000 ($933.33) (during COVID). For 100 surgeries, the extra funds required equal Rs. 50,00,000 ($66,666.67), which is the bare minimum for the fund pool we envision. This can be replenished from recurrent donors, campaign funds, crowdsourcing, and corporations with corporate social responsibility (CSR).

This model utilizes frugal innovation, with local sourcing of materials and products without reusing consumables. As seen in Table 2, the average cardiac surgery cost using the PPP model is approximately one-third the cost of cardiac surgery on mission trips and 20 times less costly than the average cardiac surgery cost in a US hospital.^32–34^ Our model utilizes a triage system, which allows us to operate on patients with correctable lesions according to RACHS 1-4. As a result, additional resources, such as conduits, are kept to a minimum, allowing us to minimize the cost of each procedure as delineated above.

**Table 2.** Average Cost Cardiac Surgery Using Different Models.

	PPP model	US hospital	Mission trip
Average cardiac surgery costs	$2400	$51,300	$6800

Abbreviations: PPP, public–private partnership; US, United States.

Currently, our target of 100 free cases per year has not been achieved due to the lack of funding and quantity of recurrent donors. With the ongoing COVID-19 pandemic, donor fatigue may be a contributory factor. However, as the infrastructure and trained team are in place, sustaining a PPP surgical cardiac program seems an achievable goal.

Table 3 outlines the costs for various cardiac procedures prior to COVID-19 and costs after the start of the pandemic. The current cost difference is due to the goods and services tax. The table shows the average costs for the various surgeries, which depend on the complexity, need for implants or special drugs, and disposables among other variables.

**Table 3.** Average Cost for Cardiac Procedures Pre-COVID and COVID Eras.^a^

Surgeries	Cost Breakdown 2014–2019 Rs/$	Cost Breakdown 2019–2021 Rs/$
Atrial septal defect closure w/autologous pericardial patch	98,000 + 4000 ($1306.67 + $53.33)	1,15,000 + 3000 ($1533.33 + $40)
Ventricular septal defect w /PTFE patch closure	97,000 + 3000 ($1293.33 + $40)	1,10,000 + 7000 ($1466.67 + $93.33)
Ventricular septal defect with pulmonary hypertension double flap PTFE patch closure	1,13,000 ($1506.67)	1,20,000 ($1600)
Tetralogy of Fallot intracardiac repair	1,13,000 + 4000 ($1506.67 + $53.33)	1,60,000 + 7000 ($2133.33 + $93.33)

Abbreviations: PTFE, polytetrafluoroethylene; Rs, Indian Rupee; USD, US dollar.

^a^ Exchange rate: 1 USD ∼ 75 Rs.

Table 4 outlines the cost for an average surgery, with a breakdown detailing the percentage of the total cost being allocated to each portion of the procedure. Most of the costs are allocated to drugs and disposables, for which the prices have increased from Rs. 60,000 to Rs. 70,000 ($800-$933.33), and hospital staff and professional services, for which the costs have remained unchanged despite COVID.

**Table 4.** Average Cost for Surgery With Breakdowns.^a^

Tasks	2014-2019 Pre-COVID Cost Rs/$	2019-2021 During COVID Cost Rs/$
Camp	1000 ($13.33)	1000 ($13.33)
Travel & Shelter (3.125%)	4000 ($53.33)	4000 ($53.33)
Drugs & Disposables (37.5%)	60,000 ($800)	70,000 ($933.33)
Investigations (12.5%)	20,000 ($266.67)	25,000 ($333.33)
Preparation before surgery (15.625%)	25,000 ($333.33)	30,000 ($400)
Hospital staff & professional services (31.25%)	50,000 ($666.67)	50,000 ($666.67)
Total	1,60,000 ($2133.33)	1,80,000 ($2400)

Abbreviations: Rs, Indian Rupee; USD, US dollar.

^a^ Exchange rate: 1 USD ∼ 75 Rs.

The cost breakdown can be divided as follows: camp, travel and shelter, drugs and disposables, investigation charges, hospital charges, professional charges, and hospital charges. The cost of holding one camp is Rs. 30,000 ($400), which is distributed among all patients waiting for surgery. For example, 50 patients were waiting for surgery in one camp. Thirty surgeries were required to be done with the amount donated within three months. Therefore, since 10 surgeries were performed per month, the holding charges for the camp totaled Rs. 1000 ($13.33) per patient. Travel and accommodation equal approximately 3% of the total costs. Travel typically includes train or bus for the family and shelter includes food for the family for the entire length of stay. This is a discounted cost due to exemptions awarded to the hospital for its services for the less affluent.

Drugs and disposables make up approximately 40% of the costs. To decrease costs, consumables are purchased in bulk either from the hospital or from a local supplier. Local suppliers allow for lower prices compared to when sourced from abroad. Indigenization and innovation must be encouraged, as resources can be obtained at costs less than 10% of the imported prices. Brazil has already taken steps toward this direction and has begun manufacturing and utilizing their materials locally.^35^ To maintain lower costs for drugs and disposables, LMICs must consider greater investment and encouragement of the medical industry.^31^ Next, investigation charges make up 12% of the finances. It is necessary to streamline the network of referral doctors, to whom the patients can go once the operation has successfully concluded. Investigations, which include echocardiograms, are charged at a discounted cost. Critical risk factors must be studied at every stage, including preoperative, perioperative, and postoperative time points, and addressed accordingly to maximize the success of the operation, especially as routine medical care may be difficult to come by in LMICs.^24^

Hospital staff and professional services charges make up 30% of the costs, which provide income for the staff who take care of the patient. For staff who work permanently on the care team, a total of Rs. 1,00,000 ($1333.33) is paid per month. The current members of the cardiac care team at PHC include two cardiac surgeons, one anesthesiologist, four pediatric specialists, one interventional cardiologist, ten nurses, three perfusionists, one occupational therapy technician, three intensivists, two physician assistants, one case manager, and many volunteers. There are some who are paid in a case-by-case scenario, in which they receive Rs. 40,000 ($533.33) per case. These healthcare workers are typically employed at other hospitals or clinics and join the team when cases arise during their own spare time. Professional services also include consultation fees for pediatric specialty consultants.

Periodical follow-up makes up 1% of the total cost. All the operated children are referred to their primary care physician. For serious postoperative problems, they are asked to come back to PHC, and their travel and accommodation are taken care of. Over the years, the entire team has become familiar with all the children that have been operated on. Most patients have access to mobile phones either directly or through neighbors or relatives. About 85% of patients attended their follow-up appointments (15% loss) with a median duration of 12 years. In the first 10 to 15 years of the program, the follow-up rate was around 60% to 70%, and patients were paid to come to their follow-ups.

Medications prescribed long-term are also covered by the fund. In general, donations up to Rs. 1,00,000 ($1333.33) are used for immediate expenses. Blood and components for each surgery are donated by Voluntary Health Services (NGO). If these resources are not available, payment to other blood banks is provided to ensure the availability of the necessary resources for the operation.

### Future Plans

Under the current model, the target population is the economically challenged children of India. By utilizing funds from CMCHIS, other allocated government funds, and recurrent donors, we envision the new PPP paradigm will be suitable for other LMICs and will sustain itself through the cooperation of the public and private spheres. We hope that the PPP model will create opportunities for patients to receive necessary cardiac care, as we know that it takes a “village” to take care of all the untreated cardiac patients in LMICs. Physicians will be paid reasonably, allowing them to continue providing care to patients without the burden of their own financial stability. This model will be more sustainable than previously attempted single-funded public or private models.

The first goal of this corpus fund is to fund 100 free surgeries at the cost of approximately Rs. 1 crore (100 lakhs, $133,333.33). If the corpus fund can reach Rs. 10 crores ($1,333,333.33), just the interest alone will provide sufficient funds to finance 100 surgeries per year without having to touch the principal amount.

### Essential Features of a Complete Model

1. *Patients:* Due to the PPP model, PHC has contact with a network of Primary Health Centre doctors in different districts. They identify economically challenged children with cardiac diseases, investigate them at District Hospitals, and refer them to PHC. Often, community camps can also identify patients and waitlist them for surgery. Currently, a network of doctors that has been created through CMCHIS exists in four districts. Further expansion of the network of doctors will provide a unified community from various districts. In addition, a feedback loop allows for long-term follow-up with patients postoperatively. Recently, the government of India has emphasized the need to address the increasing burden of NCDs in the country and create an efficient and sustainable network.^36^
2. *Team:* The second feature required is a surgical team. This is the most vital component for favorable outcomes, measured in terms of decreased mortality. This team must include operating room staff, surgeons, ICU staff, nurses, anesthesiologists, and perfusionists among others. Guaranteeing a well-oiled team is the key to successful outcomes. Here, we also would like to emphasize the importance of a “spark plug” who would take ownership and grow with the program, like many others in the world.^37^
3. *Processes and training:* Programs should focus on sustainability, self-reliance, and quality improvement (QI). QI reviews should ideally be conducted quarterly to review current protocols and standard-of-care expectations. Internal audits in the form of morbidity and mortality meetings are crucial for QI efforts. Partnerships with QI committees, such as IQIC, will allow programs to keep track of and demand improvement based on data and outcomes. In our model, partnership with IQIC allowed us to improve postoperative care and outcomes.^38^
4. *Partnerships with hospitals:* The next requirement is a partnership with a hospital that has state-of-the-art infrastructure. Typically, for a minimum of 100 surgeries per year, the requirements are: five preoperative beds, six ICU beds, and five step-downward beds. The availability of catheterization labs and other pediatric specialties is ideal. Creating partnerships with other cardiac centers in LMICs may also provide an arena for QI, resource reallocation, sharing, and collaboration. Centralization of cardiac care may be the next frontier in improving health care in low-resource populations in LMICs.^11^
5. *Funds:* Lastly, a steady flow of funds is the best way to ensure sustainability. Baseline finances should be separated from maintenance funds. In addition to free operations (patients whose annual income is under Rs. 72,000 or $960), the implementation of a sliding scale payment system will allow for subsidized care to all income groups. For those whose annual income is between Rs. 72,000 to Rs. 2 lakhs ($960-$2666.67), the subsidization costs will depend on the family size. Patients in this stratification will be expected to provide as much as they can to the revolving fund. Lastly, for patients receiving greater than Rs. 2 lakhs income (>$2666.67), they will be also provided a subsidized cost based on the complexity of their cardiac condition. The goal is to provide the same quality care irrespective of income group.

In summary, this project utilized multisource financing, through PPP funding organizations (national and international), philanthropists, and corporates with CSR. For patients who can pay even a little, they will contribute to the funds of other beneficiaries. This divides the financial burden on each individual or organization, while simultaneously allowing the economically challenged children to be treated equally to the affluent. It is not a “rob Peter to pay Paul” model. These are willing contributions from society for the underprivileged. This project has proved that this is possible.

## Limitations

The first limitation is that patient demand is greater than the currently available care. This must be addressed with triage based on presentation (asymptomatic/emergent), complexity (single operation or multiple procedures), and comorbidities (malnourishment, infections, etc). Our experience has shown that SOCAN is a pilot sample that can be replicated. For India, the current population stands at 1.39 billion. The WHO standards define optimal cardiac care in a high-income country as one cardiac center per two million population performing 300 to 500 cases annually which translates to 700 centers (approx.) to cater to the cardiac patients.^39^ Government funding alone may not be enough. As such, this new model of PPP may provide additional help for economically challenged children.

The next limitation is the slow improvement of surgical skills. A rapid improvement in surgical skills, techniques, perioperative care, and mortality rate requires a large volume of cases.^7^ Increasing volumes necessitate increased staff, more training, and resources. As such, this is often difficult to achieve at the beginning of grassroots programs. Although it is commonly believed that a minimum of 150 cases is needed for a pediatric heart surgeon to maintain skills, it is not always possible for a grassroots program, especially in LMICs. Our model provides surgery for low-risk, RACHS 1-4 categories. It has been shown that volume was not associated with mortality for low complexity cases.^40^ We believe that at a grassroots level, it is better to take on fewer cases than to decrease the quality of each operation. However, once the processes are set and an adequate team is in place (like we have in phase three of the program), increased volume and economies of scale will further refine the team and lower the cost of surgery.

The present surgical team has two surgeons who have 45 years and 20 years of experience, respectively. The senior surgeon passed through several high-volume centers, and the latter is a current Professor and Head of the Department of Pediatric Cardiothoracic Surgery at the Institute of Child Health and Hospital for Children. The cardiac anesthesiologist and chief intensivist are at present the Professor of the Department of Emergency Medicine in Kilpauk Medical College Hospital. All three have already visited Boston Children's Hospital as observers and are in regular contact with the faculty there.

The lack of state-of-the-art infrastructure in LMICs along with difficult financing poses another hurdle. This is the major hurdle in most resource-limited LMICs, where unlike in India, has no government support. This continues to perpetuate the cycle of resource-lacking cardiac programs to care for the increasing number of cardiac patients globally. This is where this model may not work. In Chennai, there are several government and private hospitals with good infrastructure. With increases in volume, the current core surgical team can support a central training center. In India, there are several cardiac centers that exist for paying patients; therefore, the goal is to sustain the unique network Cardiac Network for the Less Affluent. The creation of a stable revolving fund would help.

Finally, many LMICs may not have a “spark plug” locally as described in our model. Dr SS has been a “spark plug” who worked tirelessly for over three decades to create the present sustainable model that can be replicated in large LMICs like India.

## Conclusion

In summary, for the highest probability of success, these are the most important to secure: a trained, efficient, and independent surgical team, state-of-the-art infrastructure, and a corpus fund in this order. Compromise should never exist within the resources and quality of care divided into each case; rather the compromise should be in the number of cases that can be taken. To add, the quality of care for simple and complex cases, despite the difference in costs, should also be equal. Much like the bond between the surgical team and patient, the bond between the surgical team and the donor must also be strong. Their goals of offering the best care to the underserved with dignity are one and the same. We believe that this PPP model is a sustainable model, which may be replicable in LMICs that have some access to government support to start with.

## Abbreviations

CHD: congenital heart disease
CMCHIS: Chief Minister's Comprehensive Health Insurance Scheme
CSR: corporate social responsibility
H4H: Hearts 4 Hearts Fund
ICU: intensive care unit
IQIC: International Quality Improvement Collaborative
LMIC: low- and middle-income country
NCD: noncommunicable disease
NGO: nongovernmental organization
PHC: Public Health Centre Hospital
PPP: public–private partnership
QI: Quality improvement
RACHS: Risk Adjusted Congenital Heart Surgery Score
Rs: Indian Rupees
SOCAN: Solution for Cardiac Affected Needy
USD: United States Dollar
WHO: World Health Organization
